# Spatio–Temporal Relationship and Evolvement of Socioeconomic Factors and PM_2.5_ in China During 1998–2016

**DOI:** 10.3390/ijerph16071149

**Published:** 2019-03-30

**Authors:** Yi Yang, Jie Li, Guobin Zhu, Qiangqiang Yuan

**Affiliations:** 1 School of Remote Sensing and Information Engineering, Wuhan University, Wuhan 430079, China; yyi@whu.edu.cn; 2 School of Geodesy and Geomatics, Wuhan University, Wuhan 430079, China; yqiang86@gmail.com; 3 Collaborative Innovation Center of Geospatial Technology, Wuhan University, Wuhan 430079, China

**Keywords:** PM_2.5_ concentration, socioeconomic factors, Bivariate Moran’s I, spatial lag model

## Abstract

A comprehensive understanding of the relationships between PM_2.5_ concentration and socioeconomic factors provides new insight into environmental management decision-making for sustainable development. In order to identify the contributions of socioeconomic development to PM_2.5_, their spatial interaction and temporal variation of long time series are analyzed in this paper. Unary linear regression method, Spearman’s rank and bivariate Moran’s I methods were used to investigate spatio–temporal variations and relationships of socioeconomic factors and PM_2.5_ concentration in 31 provinces of China during the period of 1998–2016. Spatial spillover effect of PM_2.5_ concentration and the impact of socioeconomic factors on PM_2.5_ concentration were analyzed by spatial lag model. Results demonstrated that PM_2.5_ concentration in most provinces of China increased rapidly along with the increase of socioeconomic factors, while PM_2.5_ presented a slow growth trend in Southwest China and a descending trend in Northwest China along with the increase of socioeconomic factors. Long time series analysis revealed the relationships between PM_2.5_ concentration and four socioeconomic factors. PM_2.5_ concentration was significantly positive spatial correlated with GDP per capita, industrial added value and private car ownership, while urban population density appeared a negative spatial correlation since 2006. GDP per capita and industrial added values were the most important factors to increase PM_2.5_, followed by private car ownership and urban population density. The findings of the study revealed spatial spillover effects of PM_2.5_ between different provinces, and can provide a theoretical basis for sustainable development and environmental protection.

## 1. Introduction

The rapid development of China’s economy in recent decades has caused serious environmental pollution, among which atmospheric pollution is particularly serious [1,2]. The frequent haze weather across the country has seriously affected the urban environment [3,4] and the physical and mental health of residents [5,6,7]. The main pollutant forming haze weather is fine particulate matter with a diameter of less than 2.5 μm (PM_2.5_). PM_2.5_ can reduce visibility. It is harmful to people’s life, especially in health effects [8,9], so PM_2.5_ pollution has become a research hotspot. The previous studies mainly involved two aspects, namely micro aspects and macro aspects. The micro aspects focus mainly on chemical components [10,11,12] and physical and mental health effects of PM_2.5_ [13,14,15,16], etc. The macro aspects focus mainly on the influencing factors [17,18], spatiotemporal variations and distribution about PM_2.5_ [19,20], etc. Simultaneously, PM_2.5_ pollution has also hampered economic development [21]. Hence, a clear understanding of the PM_2.5_ pollution problem benefits from the research on the spatial relationships between PM_2.5_ and social economy, which can be assisted in adopting more effective methods to improve air quality.

China’s economy has entered a period of rapid development and various industries have witnessed rapid development since the “reform and opening up”. Meanwhile, many pollution sources have been increased [22,23]. Some human activities [24], such as industrial emissions, motor vehicle emissions, coal burning [25], fossil fuel burning and outdoor biomass burning and so on [26,27], produced emissions of elemental carbon (EC), organic mass (OM), inorganic ions, metal elements and secondary aerosol precursors [28], resulting in increased PM_2.5_ concentration. So, reducing these human activities may be important for controlling China’s PM_2.5_ levels, which in turn reduced the impact on the environment, economy and health caused by PM_2.5_ pollution [29]. Some socioeconomic factors can be used to reflect PM_2.5_ pollution and control pollution sources. For example, more energy consumption and emissions could be caused by higher population density [30], and motor vehicle exhaust (CO, NO, and SO_2_) also results in increased PM_2.5_ [31]. In this paper, four controllable socioeconomic factors are selected to quantify the relationships between socioeconomic development and PM_2.5_, namely GDP per capita, industrial added values, urban population density and private car ownership [17,18,32]. However, the imbalance of urbanization and economic development in China, in addition the influence of other natural factors, had resulted in spatial heterogeneity of PM_2.5_ pollution [33,34]. Therefore, some scholars put forward policy suggestions that were appropriate to local conditions for reducing the emission of PM_2.5_ in different regions [35]. Some scholars pointed out that the influence of economic urbanization and coal consumption on PM_2.5_ concentration were greater than population urbanization [36]. In China, the relationships between PM_2.5_ concentration and economic development shows an environmental Kuznets curve (EKC) of inverted U-shape [37]. Industrial atmospheric pollutants, the proportion of primary and secondary industry to GDP, population density and meteorological condition had great contributed to PM_2.5_ concentration [20,38]. PM_2.5_ of Asian and African countries had a significantly positive correlation with urbanization [39]. Although the relationship between PM_2.5_ and socioeconomic factors was investigated in many literatures, few literatures analyzed the spatial correlation between PM_2.5_ and socioeconomic factors by bivariate spatial correlation analysis method, especially from the perspective of long time series.

The spatio-temporal variations of PM_2.5_ concentration and socioeconomic factors, their traditional statistical relationships, spatial statistical relationships and spatial spillover effect of PM_2.5_ concentration were examined in this research using the multisource data of 31 provinces in China from 1998 to 2016. The findings in this study will contribute to a thorough understanding of the spatial relationships between PM_2.5_ concentration and socioeconomic factors in China, and will provide auxiliary decision support for urban sustainability and policy efficiency.

## 2. Materials and Methods

### 2.1. Data

At the website of Dalhousie University, the global surface PM_2.5_ concentration dataset that was estimated by GEOS-Chem chemical transport model combined with the aerosol optical depth (AOD) were provided by the Atmospheric Composition Analysis Group (http://fizz.phys.dal.ca/~atmos/martin/?page_id=140). The NASA MODIS, MISR, and SeaWIFS satellite instruments were used to retrieve the AOD. The global surface PM_2.5_ concentration dataset was calibrated based on global ground PM_2.5_ observations using geographically weighted regression (GWR) [40,41]. The annual average PM_2.5_ data in 31 provinces of China at a resolution of 0.1° × 0.1° from 1998 to 2016 in this study were extracted from this dataset by ARCGIS10.3 software (ESRI Inc., Redlands, CA, USA).

The socioeconomic data were obtained from the National Bureau of Statistics of the People’s Republic of China (http://data.stats.gov.cn/). In this study, four major socioeconomic factors were collected in 31 provinces of Mainland China during 1998–2016, namely GDP per capita (GDPP), industrial added values (IVA), urban population density (UPD) and private car ownership (PCO). In order to eliminate the influence of dimension, z-scores were used to standard all factors and variables.

### 2.2. Methods

Five methods were used in this paper, namely unary linear regression model, Spearman’s rank correlation analysis, univariate spatial autocorrelation, bivariate spatial correlation analysis and spatial regression analysis. The main methods were analyzed in detail below.

#### 2.2.1. Unary Linear Regression Model

In order to analyze the temporal trend, the slope of socioeconomic factors and PM_2.5_ concentration were calculated by using unary linear regression model. The slope is expressed as:

(1) $$Slope=\frac{\sum_{t=1}^{T}t\cdot Y_{t}-\frac{1}{T}\left(\sum_{t=1}^{T}t\right)\left(\sum_{t=1}^{T}Y_{t}\right)}{\sum_{t=1}^{T}t^{2}-\frac{1}{T}{\left(\sum_{t=1}^{T}t\right)}^{2}},$$

where slope is the trend gradient, $Y_{t}$ denotes the variable (PM_2.5_ concentration or GDPP or IVA or UPD or PCO) in the *t*-th year, T is the study period of 1998–2016. A positive (negative) slope means that the variable increases (decreases) over the years. The greater the absolute value of the slope, the faster the increase or decrease of speed.

#### 2.2.2. The Univariate Spatial Autocorrelation Analysis

Moran’s I, as the most commonly used indicator of global spatial autocorrelation, was initially suggested by Moran [42]. In essence, it represents the cross product statistics of a variable and its spatial lag. The degree to which the feature values of a position are similar or different from those of its spatial neighbors is measured by spatial autocorrelation. The global spatial association of PM_2.5_ concentration across China was explored by global Moran’s I in this paper. To explore the local spatial association (spatial clustering or spatial dispersion) in adjacent provinces, we chose a local indicator of spatial association (LISA) [43] as the analysis method. The global Moran’s I and local Moran’s I are calculated by:

(2) $$I=\frac{n}{\sum_{i=1}^{n}\sum_{j=1}^{n}w_{ij}}\frac{\sum_{i=1}^{n}\sum_{j=1}^{n}w_{ij}\left(x_{i}-\bar{x}\right)\left(x_{j}-\bar{x}\right)}{\sum_{i=1}^{n}{\left(x_{i}-\bar{x}\right)}^{2}}$$

(3) $$I_{i}=\frac{x_{i}-\bar{x}}{\sigma^{2}}\sum_{j=1}^{n}w_{ij}\left(\frac{x_{j}-\bar{x}}{\sigma }\right),$$

where I stands for global Moran’s I for the whole study region, $I_{i}$ is the Moran’s I for province *i*, $x_{i}$ donates PM_2.5_ concentration at province *i*, $x_{j}$ donates PM_2.5_ concentration at all the other provinces (where

j≠i).

Also, $\bar{x}$ is the mean PM_2.5_ concentration of 31 provinces in China, n represents the total number of provinces. σ is the standard deviation of the PM_2.5_ concentration of 31 provinces. $w_{ij}$ is the spatial weight matrix, representing province i is adjacent to province j, neighboring provinces were 1 and non-adjacent provinces were 0. The values of I or $I_{i}$ ranged from −1 to 1. A positive (negative) I or $I_{i}$ value indicates positive (negative) spatial autocorrelation in the provinces. Positive autocorrelation indicates that provinces with similar PM_2.5_ concentration are closely distributed in space, whereas negative spatial autocorrelation indicates that PM_2.5_ concentration of neighboring provinces are dissimilar. A zero I or $I_{i}$ value indicates a random spatial pattern. The size of the absolute value of I or $I_{i}$ can reflect the strength of the spatial correlation.

#### 2.2.3. The Bivariate Spatial Correlation Analysis

The spatial correlation between PM_2.5_ and socioeconomic factors were examined by global bivariate Moran’s I and local bivariate Moran’s I. Global bivariate Moran’s I reflects the global spatial associations between PM_2.5_ and another variable (GDPP, IVA, UPD or PCO) across the whole region, whereas local bivariate Moran’s I explores the local spatial correlations within different provinces [44,45,46]. Global bivariate Moran’s I and local bivariate Moran’s I are given by:

(4) $$I_{xy}=\frac{n\sum_{i=1}^{n}\sum_{j\neq i}^{n}w_{ij}Z_{i}^{x}Z_{j}^{y}}{\left(n-1\right)\sum_{i=1}^{n}\sum_{j\neq i}^{n}w_{ij}}$$

(5) $$I_{xy}^{i}=Z_{i}^{x}\sum_{j=1,j\neq i}^{n}w_{ij}Z_{j}^{y},$$

where $I_{xy}$ is the global bivariate Moran’s I, and $I_{xy}^{i}$ is the local bivariate Moran’s I in province i. n is the total number of provinces, and $w_{ij}$ is the queen contiguity weight matrix. $Z_{i}^{x}$ is the standardized z-scores of PM_2.5_ concentration in the *i*-th province, $Z_{j}^{y}$ is the standardized z-scores of socioeconomic factors (GDPP, IVA, UPD or PCO) in the *j*-th province. The values of $I_{xy}$ or $I_{xy}^{i}$ is in the range [−1,1]. The values of $I_{xy}$ or $I_{xy}^{i}$ greater than 0, less than 0, equal to 0 indicate positive spatial correlation, negative spatial correlation, or no correlation between PM_2.5_ concentration and socioeconomic factors, respectively. The size of the absolute value of $I_{xy}$ or $I_{xy}^{i}$ can reflect the strength of the spatial correlation.

#### 2.2.4. The Spatial Regression Model

Spatial lag model (SLM) and spatial error model (SEM) were based on the ordinary least squares (OLS) [47,48]. SLM can be used to explore whether PM_2.5_ concentration diffuses in one province, whereas SEM can be used to interpret the dependence of spatial error [44]. The SLM and SEM can be defined as follows:

(6) $$Y_{it}=\alpha +\rho wY_{it}+\beta_{1}x_{GDPP}+\beta_{2}x_{IVA}+\beta_{3}x_{UPD}+\beta_{4}x_{PCO}+\varepsilon$$

(7) $$Y_{it}=\alpha +\beta_{1}x_{GDPP}+\beta_{2}x_{IVA}+\beta_{3}x_{UPD}+\beta_{4}x_{PCO}+\lambda w_{\mu }+\varepsilon ,$$

where $Y_{it}$ denotes PM_2.5_ concentration in province i in the *t*-th year, α represents a constant term. $\beta_{1}$, $\beta_{2}$, $\beta_{3}$, and $\beta_{4}$ are the parameters to reveal the correlations between PM_2.5_ and GDPP, IVA, UPD, and PCO, respectively. $wY_{it}$ is a spatial lag-dependent variable vector, it reflects the endogenous interaction effects among $Y_{it}$, ρ is a spatial regression coefficient that denotes the spatial dependence of the sample observations. $w_{\mu }$ reflects the interaction effects among the disturbance term of different provinces. The spatial autoregressive coefficient λ denotes the spatial dependence of the residuals; ε is the random error term, μ represents the spatially autoregressive error terms.

In order to determine whether SLM or SEM is more suitable for the simulation of PM_2.5_, a Lagrange multiplier (LM) test and robust Lagrange multiplier (RLM) test should be estimated by the OLS. Anselin et al. proposed the criterion that if SLM-LM and SEM-LM are not significant, the OLS model was selected as the final model. If SLM-LM is significant and SEM-LM is not significant, SLM will be selected, and vice versa for SEM; if both SLM-LM and SEM-LM are insignificant, SLM-RLM is significant but SEM-RLM is not significant, SLM will be selected; if both SLM-LM and SEM-LM are insignificant, SEM-RLM is significant but SLM-RLM is not significant, SEM will be selected [46]. The univariate spatial autocorrelation analysis, the bivariate spatial correlation analysis and the spatial regression analysis were conducted in GeoDa software (GeoDa Press LLC, Chicago, IL, USA), and we chose queen contiguity weight matrix in GeoDa software.

## 3. Results

### 3.1. The Spatial Distribution of Socioeconomic Factors and PM_2.5_ in China

**Figure 1:**
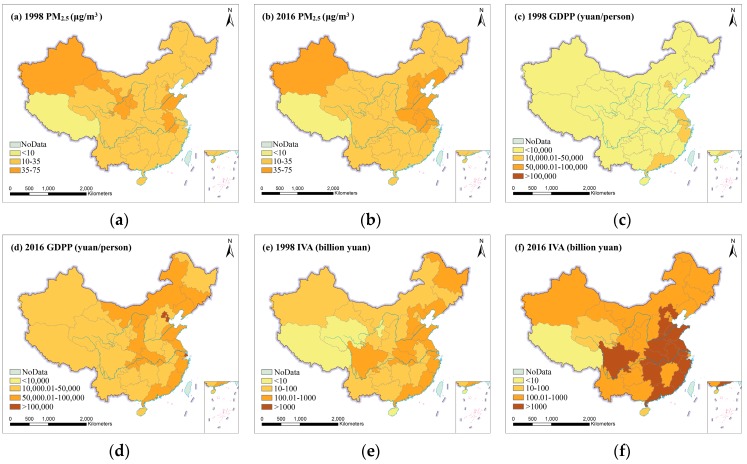

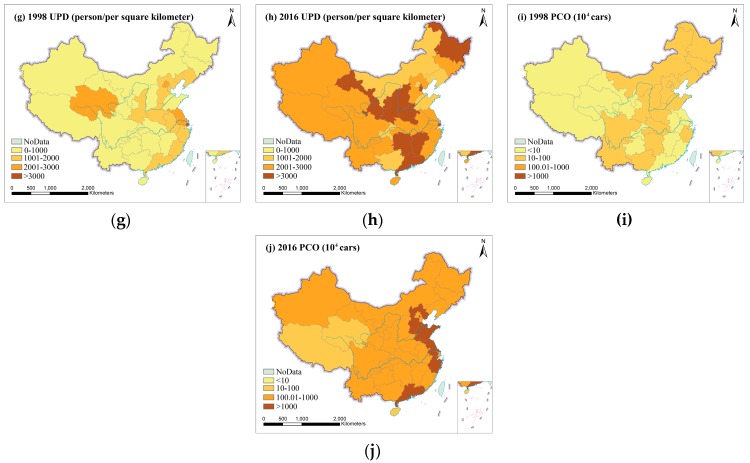
Spatial distribution of socioeconomic factors and PM_2.5_ in 31 provinces of China in 1998 and 2016. (**a**) 1998 PM_2.5_, (**b**) 2016 PM_2.5_, (**c**) 1998 GDP per capita, (**d**) 2016 GDP per capita, (**e**) 1998 industrial added values, (**f**) 2016 industrial added values, (**g**) 1998 urban population density, (**h**) 2016 urban population density, (**i**) 1998 private car ownership, (**j**) 2016 private car ownership.

From Figure 1, 31 provinces in China were classified and mapped according to the values of PM_2.5_ and socioeconomic factors. From Figure 1a, it could be found that PM_2.5_ concentration only in Tibet met the WHO Air Quality Guideline (AQG) level (10 μg/m^3^) in 1998. PM_2.5_ concentration in most provinces were observed between 10 μg/m^3^ and 35 μg/m^3^. Furthermore, PM_2.5_ concentration in some provinces, such as Tianjin, Anhui, Shandong, Gansu, Ningxia, and Xinjiang, were found to be higher than 35 μg/m^3^. From Figure 1b, in 2016, obvious changes mainly occurred in some provinces of China. For example, PM_2.5_ concentration increased obviously (>35 μg/m^3^) in Liaoning, Beijing Hebei, Jiangsu, Shanghai and Henan; however, PM_2.5_ concentrations in Gansu and Ningxia were found to have fallen below 35 μg/m^3^. The distributions of socioeconomic factors were similar to the distribution of PM_2.5_ both in 1998 and 2016 generally. From Figure 1c,d, provinces with GDPP below 10,000 yuan accounted for more than 80% in 1998. Obviously, GDPP in all provinces was higher than 10,000 yuan in 2016, some of which had a GDPP of more than 100,000 yuan, such as Shanghai, Beijing and Tianjin. Figure 1e,f show that IVA increased rapidly in most provinces of China, especially in North China, East China, Central China and Northeast China. The UPD exceeded 2000 person/per square kilometer only in Shanghai, Jiangsu, Beijing and Qinghai in 1998 (Figure 1g). In 2016, the UPD in most provinces was higher than 2000 person/per square kilometer, and some provinces even exceeded 3000 person/per square kilometer (Figure 1h). From Figure 1i,j, we could find that PCO in Mainland China was less than 1 million in 1998. PCO in 31 provinces was more than 1 million, except Tibet and Qinghai in 2016.

### 3.2. The Temporal Variation of Socioeconomic Factors and PM_2.5_

#### 3.2.1. The Temporal Variation of Socioeconomic Factors and PM_2.5_ in China

**Figure 2:**
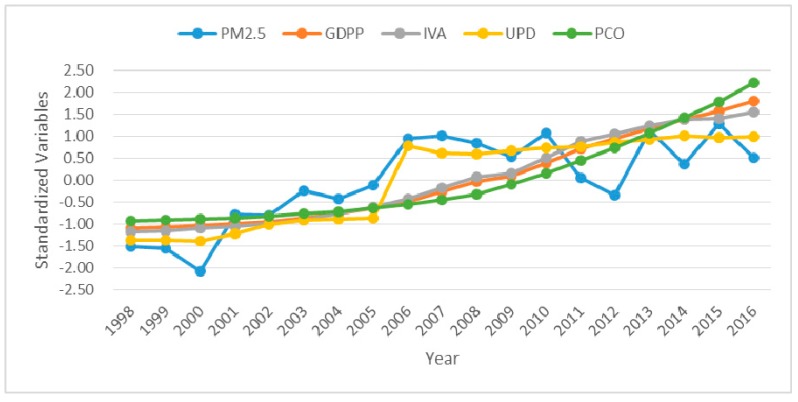
Temporal variations of standardized PM_2.5_, GDP per capita (GDPP), industrial added values (IVA), urban population density (UPD) and private car ownership (PCO) in Mainland China Figure 1998 to 2016.

Annual data on socioeconomic factors and PM_2.5_ concentration in 1998 and 2016 were counted in Table 1, and Table 2 showed the temporal variation trend (the fitted slope) of 1998–2016. In 1998, PM_2.5_, GDPP, IVA, UPD and PCO in Mainland China were 23.97 μg/m^3^, 6860 yuan/person, 3413.49 billion, 459 person/per square kilometer and 4.24 million private cars, respectively; and reached to 29.68 μg/m^3^, 53,935 yuan/person, 24787.78 billion, 2408 person/per square kilometer and 163.3 million private cars in 2016, respectively. The fitted slope of PM_2.5_, GDPP, IVA, UPD and PCO in Mainland China were 0.138, 0.173, 0.174, 0.164 and 0.165, respectively. These indicated that GDPP, IVA, UPD, PCO and PM_2.5_ generally increased from 1998 to 2016, but the increased trend of IVA, GDPP, PCO and UPD was faster than the increased trend of PM_2.5_. Figure 2 showed their temporal variations intuitively. It could be found that GDPP, IVA, UPD and PCO in Mainland China showed an increase trend gradually in 1998–2016. The PM_2.5_ concentration also increased generally but began to fluctuate sharply from 2010. It indicated that the increasing trend of PM_2.5_ concentration was similar to that of GDPP, IVA, UPD and PCO in 1998–2016, and this increasing trend was significant, especially before 2006.

#### 3.2.2. The Temporal Variation of Socioeconomic Factors and PM_2.5_ in the Seven Geographical Subareas

**Figure 3:**
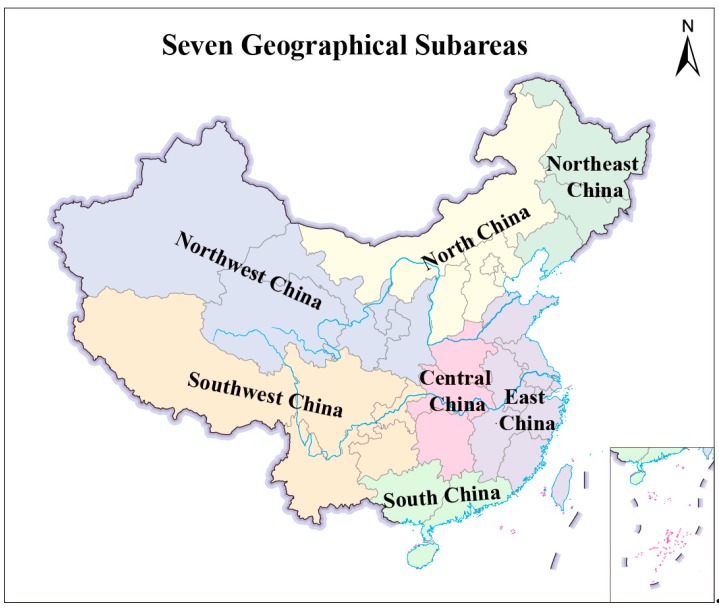
Seven regions of China.

**Figure 4:**
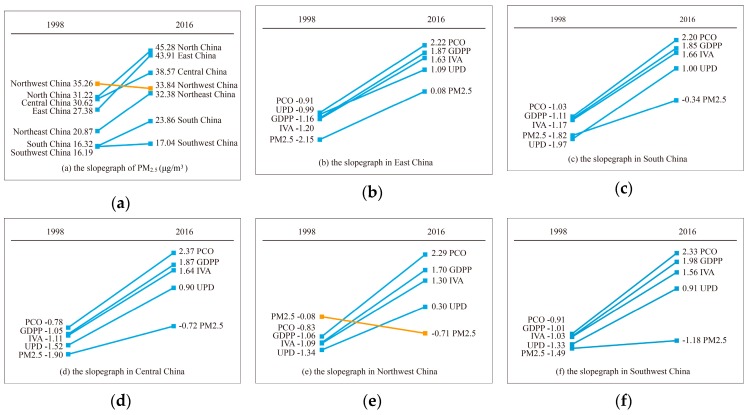

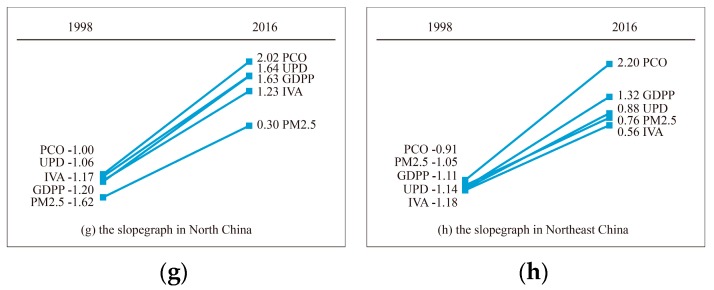
The slopegraph for explaining standardized annual mean PM_2.5_ concentration, standardized socioeconomic factors changes 1998 vs. 2016, (**a**) the slopegraph of PM_2.5_ in the seven geographical subareas, (**b**) the slopegraph of PM_2.5_ and socioeconomic factors in East China, (**c**) South China, (**d**) Central China, (**e**) Northwest China, (**f**) Southwest China, (**g**) North China, and (**h**) Northeast China.

The seven regions of China are showed in Figure 3. From Table 1, in 1998, the GDPP and UPD in East China and North China, the IVA in East China, and the PCO in North China and Central China were far higher than other geographical subareas. The GDPP, IVA, UPD, and PCO in Northwest China were relatively lower. However, Northwest China had the highest PM_2.5_ concentration (35.26 μg/m^3^). In 2016, compared with other regions, GDPP in East China and North China, and IVA in East China were still relatively higher. Notably, East China had the most private cars. Central China was the most densely populated. North China became the region with the highest PM_2.5_ concentration, followed by East China. To better analyze the variation of PM_2.5_ (GDPP, IVA, UPD or PCO) in seven sub-regions, this slopegraph in Figure 4 can be used to show the increases/decreases between just two fixed points (1998 and 2016) for different factors. Most importantly, slopegraph focused on the overall macro change between two periods points, not changes in each year or intervening period. Slopegraph is a great visualization method for focusing on that aspect of the macro change.

From Table 2 and Figure 4, it could be found that PM_2.5_ concentration in subareas except Northwest China and Southwest China presented an obviously increasing trend, and PM_2.5_ concentration in Southwest China increased slowly over the years. However, PM_2.5_ concentration in Northwest China presented a descending trend. Some literatures suggested that sand and dust was the major cause of affecting PM_2.5_ concentration in Northwest China [38]. The possible reason on the minus slope (−0.015) for PM_2.5_ in Northwest China may be an increase in vegetation coverage [49], and the decrease of dust events in Northern China in recent decades. The reduction of the wind speed in the northern hemisphere was the main reason for the decrease of dust event incidence [50]. The socioeconomic factors in the seven geographical subareas all presented an increasing trend, likely leading to the increase of PM_2.5_ concentration between 1998 and 2016.

#### 3.2.3. The Spatial Distribution of Temporal Trends for Socioeconomic Factors and PM_2.5_ in Different Provinces

**Figure 5:**
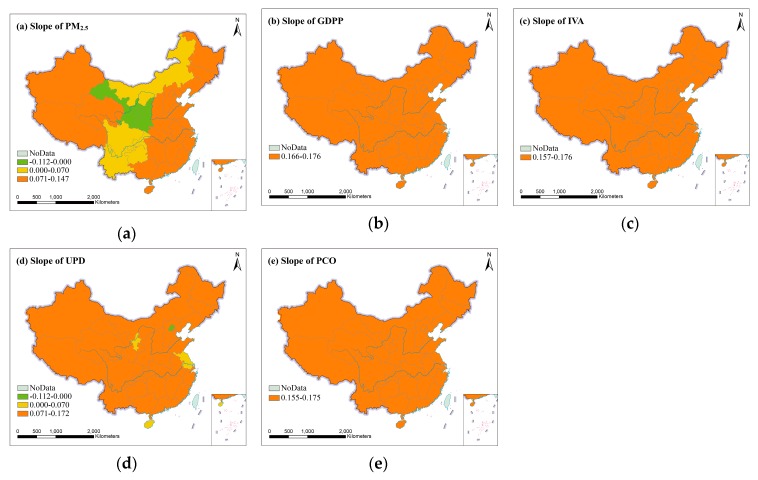
The slope of (**a**) PM_2.5_, (**b**) GDPP, (**c**) IVA, (**d**) UPD and (**e**) PCO from 1998 to 2016.

The slope values of different provinces were mapped in Figure 5. From Figure 5a, it could be found that PM_2.5_ in most provinces of China increased rapidly. The provinces with a slower growth in PM_2.5_ were mainly distributed in Inner Mongolia, Sichuan, Chongqing, Guizhou and Yunnan. On the contrary, PM_2.5_ of Gansu, Ningxia, and Shaanxi presented showed a downward trend. From Figure 5b–e, we can see that the fitted slopes of GDPP, IVA and PCO in different provinces were all more than 0.155, indicating that the increased trends of GDPP, IVA and PCO were rapid in provinces in 1998–2016. UPD increased rapidly in most provinces except Beijing, Ningxia, Jiangsu and Hainan, and the fitted slope of UPD only in Beijing was negative, this may be because of Beijing’s population control policies.

### 3.3. The Traditional Statistical Relationship between Socioeconomic Factors and PM_2.5_

#### 3.3.1. The Correlation between Socioeconomic Factors and PM_2.5_ in Mainland China

**Figure 6:**
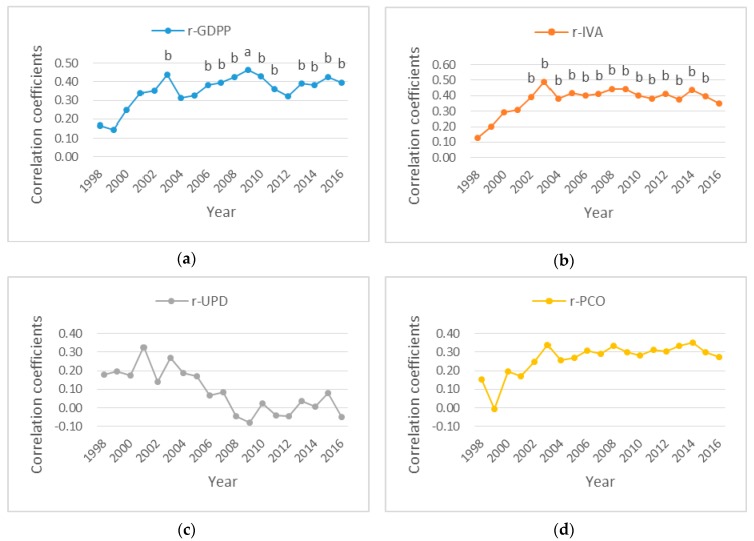
The correlation coefficients between PM_2.5_ and socioeconomic factors in Mainland China, 1998–2016. (**a**) Correlation coefficients between PM_2.5_ and GDPP. (**b**) Correlation coefficients between PM_2.5_ and IVA. (**c**) Correlation coefficients between PM_2.5_ and UPD. (**d**) Correlation coefficients between PM_2.5_ and PCO. Notes: the letters a and b above the curve point represent coefficients significant at the 1%, 5% levels, respectively. No letters above the curve point indicate insignificance.

The Spearman’s rank correlation coefficients (r-GDPP, r-IVA, r-UPD and r-PCO) between PM_2.5_ concentration and GDPP, IVA, UPD, and PCO in Mainland China in 1998–2016 were shown in Figure 6. Most of the correlation coefficients in Figure 6 were positive, indicating that PM_2.5_ was positively correlated with socioeconomic factors. From Figure 6a,b, the values of r-GDPP and r-IVA presented positive increasing trends in 1998–2003 and fluctuated around 0.4 in 2004–2016, with most of the *p*-values less than 0.05, indicating that PM_2.5_ and GDPP and IVA were significantly positively correlated during the most study years; and the correlations strengthened in 1998–2003, then appeared fluctuations in 2004–2016. From Figure 6c,d, most of the correlation coefficients were positive except for a few years. All the p-values were higher than 0.05, indicating that PM_2.5_ had a positively correlation with UPD and PCO in most years, but the correlations were not significant during the research period. The weak correlation between PM_2.5_ and PCO increased obviously before 2003. However, the correlation coefficients between PM_2.5_ and UPD presented a downward trend since 2001, indicating that the impact of UPD on PM_2.5_ was getting weaker and weaker.

#### 3.3.2. The Relationship between Socioeconomic Factors and PM_2.5_ in Provinces

**Figure 7:**
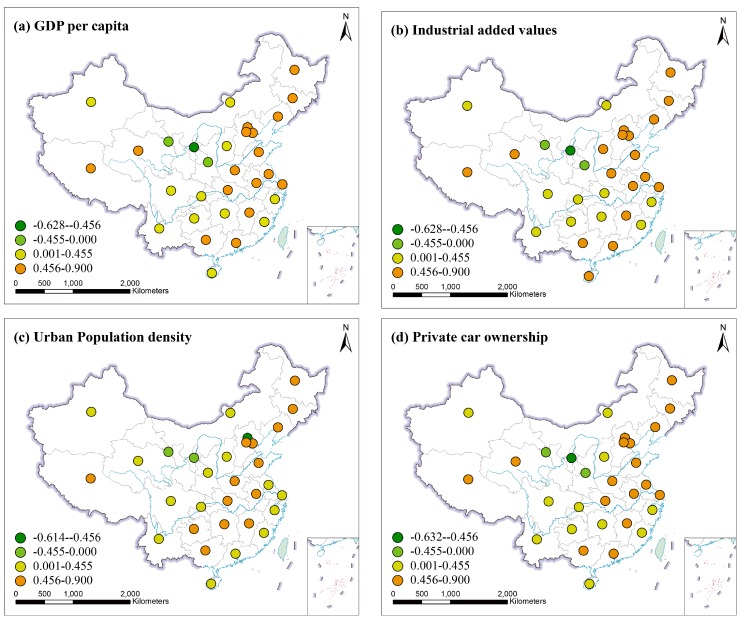
The correlation coefficient (r) values in 31 provinces. (**a**) Correlation between PM_2.5_ and GDPP. (**b**) Correlation between PM_2.5_ and IVA. (**c**) Correlation between PM_2.5_ and UPD. (**d**) Correlation between PM_2.5_ and PCO.

In the Figure 7, dark green represents a significant negative correlation, light green is a negative correlation, dark yellow means a significant positive correlation, and pale yellow represents a positive correlation. From Figure 7, PM_2.5_ in most provinces of Northeast China, North China, Central China, East China and South China showed a significantly positive correlation with GDPP, IVA, and PCO. But PM_2.5_ only in Ningxia was significantly negatively correlated with GDPP, IVA, and PCO. Most provinces of Northeast China, North China, Central China and East China showed significantly positive correlations between PM_2.5_ and UPD. However, PM_2.5_ had a negative correlation with UPD in Beijing, Gansu and Ningxia. Especially in Beijing, PM_2.5_ was significantly negative correlated with UPD. These indicated that socioeconomic factors have contributed to the increased PM_2.5_ in most provinces. But the impacts of GDPP, IVA, and PCO on PM_2.5_ in Shaanxi, Gansu and Ningxia were negative; and PM_2.5_ of Gansu, Ningxia and Beijing were affected negatively by UPD. All of these illustrate the existence of spatial heterogeneity.

#### 3.3.3. The Relationship between Socioeconomic Factors and PM_2.5_ in the Geographical Subareas

**Figure 8:**
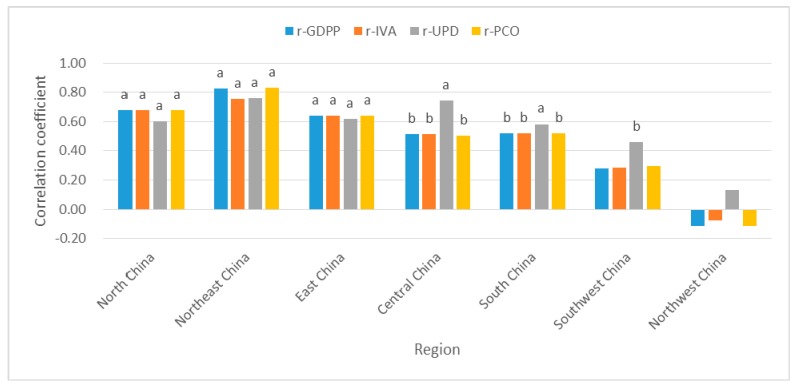
The correlation coefficient (r) values between PM_2.5_ and GDPP (IVA, UPD or PCO) in the geographical subareas. Notes: a, b represent coefficients significant at the 1%, 5% levels, respectively. No letters above the bar chart indicate insignificance.

From Figure 8, a highly significant (*p* < 0.01) positive correlation between socioeconomic factors and PM_2.5_ was observed in North China, Northeast China and East China, indicating that GDPP, IVA, UPD and PCO played a vital role in North China, Northeast China and East China. In North China, the impact of UPD on PM_2.5_ was relatively low. GDPP and PCO had a stronger effect on PM_2.5_ in Northeast China. Four socioeconomic factors had similar effects on PM_2.5_ in East China. UPD in South China had a slightly greater impact on PM_2.5_. In Central China, UPD was the major effect factor on PM_2.5_. UPD in Southwest China was the most important factor for PM_2.5_. Whether positive correlation or negative correlation, PM_2.5_ in Northwest China had no significant correlations with four influencing factors. This meant that the trend of PM_2.5_ concentration was less affected by those human activities. This was consistent with anthropogenic effects on the dust loading in East China was far higher than near desert source regions in Northwest China [50]. There were other factors which determines the PM_2.5_ trend in Northwest China, PM_2.5_ in Northwest China was mainly affected by sand and dust [38]. Previous studies have shown a positive correlation between air temperature and PM_2.5_ concentration in summer in Northwest China [51].

### 3.4. The Spatial Statistical Relationship between Socioeconomic Factors and PM_2.5_

#### 3.4.1. Global Spatial Autocorrelation of PM_2.5_

**Figure 9:**
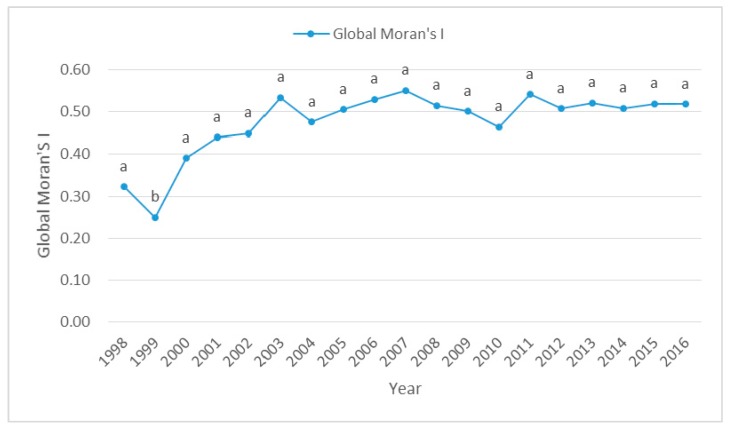
Global Moran’s I of PM_2.5_ concentration for 31 provinces, 1998–2016. Notes: a, b represent coefficients significant at the 1%, 5% levels, respectively.

From Figure 9, the global Moran’s I values of PM_2.5_ were positive at the 95% confidence level and increased over time, but fluctuated around 0.5 since 2003, indicating that PM_2.5_ exibited significantly positive spatial autocorrelation and spatial homogeneous, and spatial autocorrelation of PM_2.5_ in 31 provinces of China strengthened gradually. In other words, PM_2.5_ at one province tended to be similar to those of their neighboring provinces, the spatial spillover effect had been increasing in different provinces.

**Figure 10:**
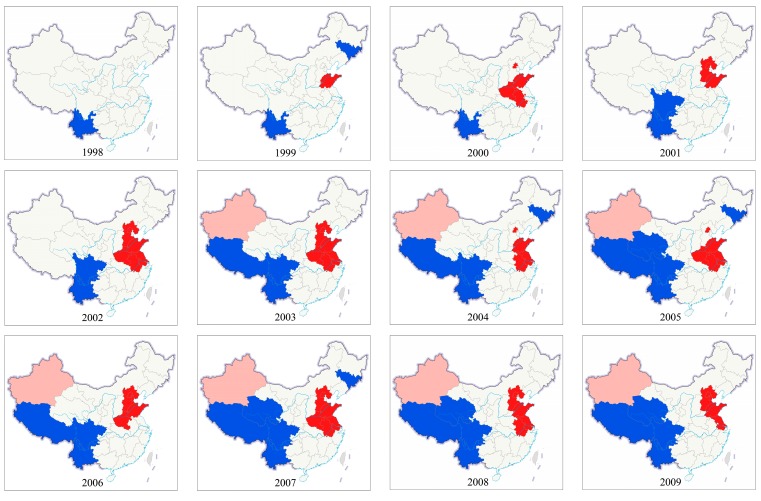

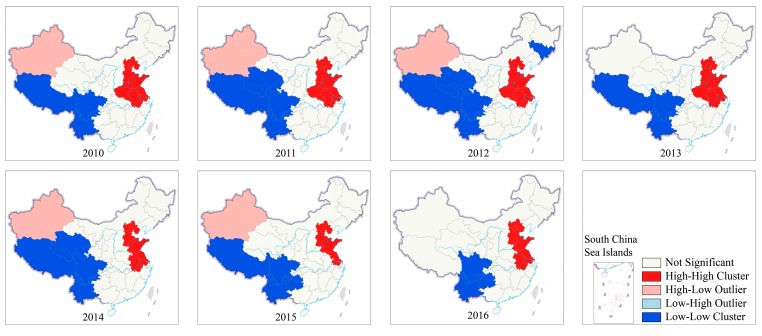
LISA cluster map of PM_2.5_ concentration for 31 provinces, 1998–2016.

In order to identify the provinces with significant spatial correlation and type of spatial clusters of PM_2.5_, LISA was calculated and mapped in Figure 10. High–high (HH) clusters means that PM_2.5_ concentration of one province and its neighbors were higher than the annual average PM_2.5_ concentration in Mainland China. While, low–low (LL) clusters refers to the provinces with low PM_2.5_ concentration being surrounded by neighbors with low PM_2.5_ concentration, whose value is lower than the annual average values. High–low (HL) outliers means that high PM_2.5_ concentration had low PM_2.5_ concentration in the neighboring provinces and vice versa for the low–high (LH) outliers. The HH and LL clusters can reflect the similar PM_2.5_ concentration clustering, indicating spatial autocorrelation of PM_2.5_ is positive; spatial dispersion of PM_2.5_ concentration is reflected in the HL and LH outliers, it indicates that PM_2.5_ concentrations have a negative spatial autocorrelation. From Figure 10, the spatial spillover effect of PM_2.5_ pollution in Southwest China, North China and East China were the most significant from 2003 to 2016. This may be because some geographic and meteorological conditions (wind speed and direction, high temperature) have caused the diffusion of particulate matter. The provinces in Southwest China and Qinghai showed an LL clustering pattern during the study period. This finding may be because of the sparse population, low development intensity, high vegetation coverage and low industrial pollution. Meanwhile, HH clustering were mostly distributed in some provinces of North China, East China, and Central China from 1999 to 2016. This could be largely attributed to intensive industries, car exhaust emissions and a sharp increase in urban population density. HL outliers were mainly distributed in Xinjiang in 2003–2015 except for 2013.

#### 3.4.2. Spatial Correlations between PM_2.5_ and Socioeconomic Factors

**Figure 11:**
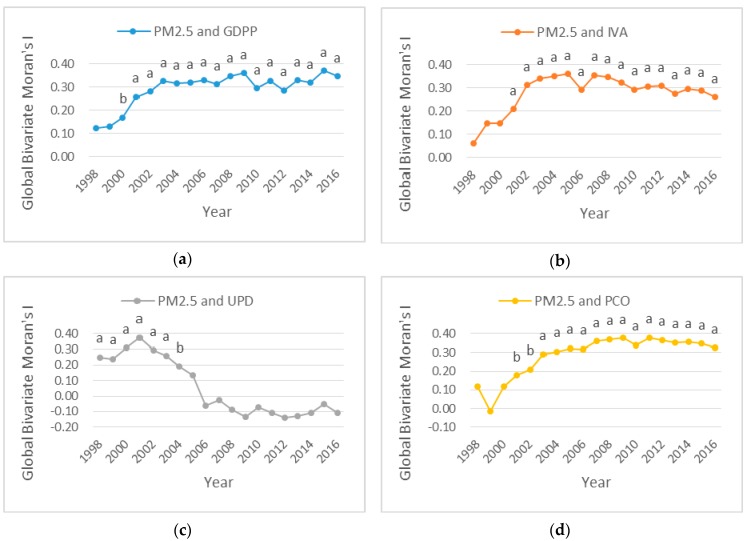
Global Bivariate Moran’s I for province-level between PM_2.5_ and socioeconomic factors in Mainland China, 1998–2016. (**a**) Global Bivariate Moran’s I between PM_2.5_ and GDPP. (**b**) Global Bivariate Moran’s I between PM_2.5_ and IVA. (**c**) Global Bivariate Moran’s I between PM_2.5_ and UPD. (**d**) Global Bivariate Moran’s I between PM_2.5_ and PCO. Notes: the letters a and b above the curve point represent coefficients significant at the 1%, 5% levels, respectively. No letters above the curve point indicate insignificance.

In this paper, global bivariate Moran’s I was used to determine if PM_2.5_ in one province were spatial correlated with socioeconomic factors of its neighbors across the study region. From Figure 11a,b,d, the global bivariate Moran’s I values presented positive growth trends at a 95% confidence level in 1998–2016. These indicated that spatial correlations between PM_2.5_ at a province and GDPP, IVA and PCO of its adjacent provinces were positive and significant, and the positive spatial correlations increased during the study period. From Figure 11c, the global bivariate Moran’s I values presented a positive increasing trend in 1998–2001, a positive decreasing trend in 2001–2005, and a negative decreasing trend in 2006–2016, with the *p*-values less than 0.05 in 1998–2004, indicating that spatial correlation between PM_2.5_ concentration at a province and UPD of its neighboring provinces was positive and significant in 1998–2004, positive but not significant in 2005, negative but not significant in 2006–2016. The spatial correlation decreased from 2001.

**Figure 12:**
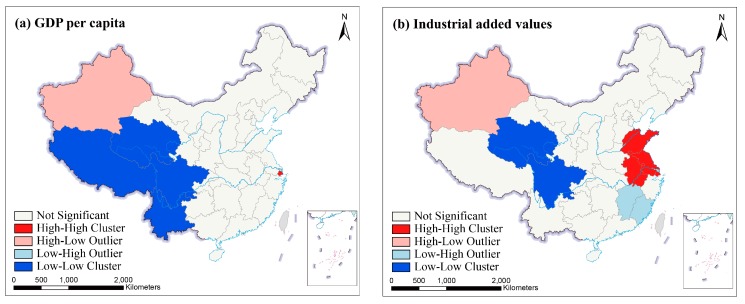

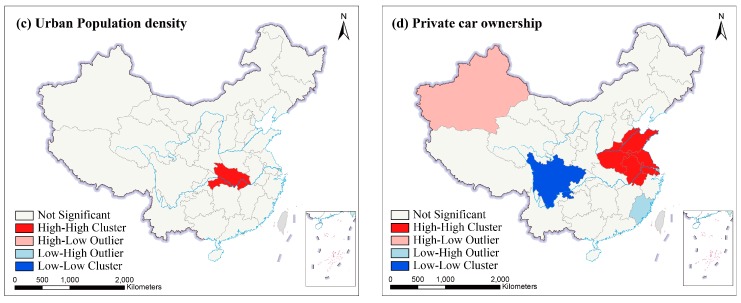
Bivariate cluster maps in Mainland China, in 2016. (**a**) Bivariate cluster map of PM_2.5_ concentration and GDPP. (**b**) Bivariate cluster map of PM_2.5_ and IVA. (**c**) Bivariate cluster map of PM_2.5_ and UPD. (**d**) Bivariate cluster map of PM_2.5_ and PCO.

As shown in Figure 12, the bivariate local Moran’s I values for PM_2.5_ and socioeconomic factors were calculated. HH clusters means that the provinces with high PM_2.5_ concentration clustered the neighboring provinces with high values of GDPP, IVA, UPD and PCO, and their values were higher than their annual average values. LL clusters means that the provinces with low PM_2.5_ concentration were near predominantly the provinces with low values of GDPP, IVA, UPD and PCO, and their values were lower than their annual average values. HL outliers occur where the neighbors of the provinces with high PM_2.5_ concentration have low GDPP, IVA, UPD and PCO. LH outliers mean that there were low values of PM_2.5_ concentration in one province, and there were high values of GDPP, IVA, UPD and PCO in the adjacent provinces. We used data from 2016 as an example to analyze the local spatial correlations between PM_2.5_ concentration and socioeconomic factors. In 2016, from Figure 12c, Hubei was the only province where appeared HH clusters of PM_2.5_ concentration and UPD. From Figure 12a,b,d, the LL clusters of PM_2.5_ concentration and GDPP (IVA or PCO) were mostly covered in some provinces of Southwest China. Shanghai appeared a HH clusters of PM_2.5_ concentration and GDPP. Shandong, Jiangsu, Shanghai and Anhui were the provinces that had a HH clusters of PM_2.5_ concentration and IVA. High PM_2.5_ concentration and high PCO clustered in Henan, Shandong, Jiangsu, Shanghai and Anhui. The place where was the HL outliers of PM_2.5_ concentration and GDPP (IVA or PCO) was Xinjiang. The LH outliers of PM_2.5_ concentration and IVA were covered in Fujian and Jiangxi. Fujian was the province that appeared a LH outliers of PM_2.5_ concentration and PCO.

### 3.5. Regression Results of the Spatial Regression Model

The analysis results of spatial autocorrelation confirmed the existence of spatial dependence of PM_2.5_, so spatial regression models were used to further confirm the spatial dependence of PM_2.5_ concentration. First, the estimated results of OLS were calculated in Table 3. LM test and RLM test were performed for the residuals of OLS regression. The values of SLM-LM and SEM-LM were significant (*p* < 0.05) except for 1999 and 2016. The values of SLM-RLM were significant (*p* < 0.1) in most years, while SEM-RLM was not significant in 1998–2016. Therefore, the SLM model was adopted. The results of spatial lag model regression in 1998–2016 were shown in Table 4. PM_2.5_ increased significantly under GDPP impact in most years (*p* < 0.1). Futhermore, IVA had also a significantly positive impact on PM_2.5_ in 2003–2010, a possible reason for this may be that some provinces (Hebei, Jiangsu and Zhejiang) had significantly increased their industrial energy consumption (>50%) in 2000–2010, resulting in the direct impact of industrialization on PM_2.5_, according to China’s Energy Statistic Yearbook (2011). But the impact of UPD and PCO on PM_2.5_ was insignificant (*p* > 0.1) in most years. The spatial autoregressive coefficient (W*PM25) were all significant (*p* < 0.01) in 1998–2016, indicating there was a significant spatial spillover effect on PM_2.5_ in adjacent provinces.

## 4. Discussion

### 4.1. Spatial Distribution and Temporal Variation of PM_2.5_ and Socioeconomic Factors

Based on four socioeconomic factors dataset and PM_2.5_ concentration dataset, this study examined the spatial distribution and relationships between socioeconomic factors and PM_2.5_ in 31 provinces of Mainland China during the period of 1998–2016. From Figure 1, provinces with high PM_2.5_ concentration have shifted from Northwest China to North China and East China since 1998, and most provinces of Northeast China, North China and East China had serious PM_2.5_ pollution in 2016. Previous studies have also shown that high PM_2.5_ concentration were mainly distributed in economically developed areas [38]. From Figure 2, the temporal variations showed that the overall increase trend of PM_2.5_ is the same as that of GDPP, IVA, UPD and PCO during 1998–2016, but PM_2.5_ exhibited a downward trend from 2006 to 2012. The reason for this phenomenon may be the implementation of sustainable development policies of energy conservation, pollutant reduction and green development proposed in the eleventh five-year plan [52]. The external cause could be meteorological factors. For example, the nitrate and secondary organic aerosols formation was greatly facilitated by high humidity [53]. Wind speed is conducive to the diffusion of PM_2.5_ [54]. The chemical reaction rate of PM_2.5_ precursor pollutants accelerates with the increase of temperature and solar radiation. [55,56]. From Table 1 and Table 2 and Figure 4, in 1998, the two regions with the highest PM_2.5_ concentration were Northwest China and North China, which were replaced by North China and East China respectively in 2016. The growth trend of GDPP, IVA, UPD, PCO and PM_2.5_ in East China, South China, Central China, North China, and Northeast China were fast. Although the growth trend of GDPP, IVA, UPD and PCO in Southwest China and Northwest China were also fast, PM_2.5_ presented a slowly growth trend in Southwest China and a descending trend in Northwest China. Previous studies had come to similar conclusions [19,50]. From Figure 5, a downward trend of PM_2.5_ presented in Gansu, Ningxia, and Shaanxi is mainly attributed to implementation of clean air policies in recent years. UPD only in Beijing showed a downward trend, which is in line with the requirements of the Beijing–Tianjin–Hebei coordination to "strictly control the increase, dredge the stock, dredge the combination" of Beijing’s population size. In addition to the policy factor of Beijing–Tianjin–Hebei cooperation, the negative growth of Beijing’s permanent population is also related to the overall trend of population returns in labor-exporting provinces.

### 4.2. The Relationships between PM_2.5_ and Socioeconomic Factors

From Figure 7, the Spearman’s rank correlation analysis indicated that four socioeconomic factors produced an increase of PM_2.5_ in most provinces of China. However, GDPP, IVA and PCO appeared a negative correlation with PM_2.5_ in Shaanxi, Gansu and Ningxia. Gansu, Ningxia and Beijing were the negative correlation between PM_2.5_ and UPD. From Figure 8, the socioeconomic factors had strong impact on PM_2.5_ concentration in North China, Northeast China and East China, but in contrast less affected PM_2.5_ concentration in Northwest China. It’s worth noting that meteorological factors and urban fugitive dust also contributed to PM_2.5_ concentration [57,58]. Soil and desert dust was the major cause of high Fe and K contents in urban fugitive dust in Northern China, and PM_2.5_ was more affected by soil dust in northern China than in southern China [59]. Furthermore, coal combustion produced fugitive dust which increased PM_2.5_ concentration. This influence was especially strong in Northern part of China [60]. In addition, it was reported that desert dust and soil dust often affected Northwest China. So, sand and dust played an important role in influencing PM_2.5_ concentration in Northwest China [38,61,62]. GDPP and IVA appeared significantly positive correlations with PM_2.5_ in most years in Figure 6a,b. While the correlation between PM_2.5_ and UPD (or PCO) was all insignificant in Figure 6c,d. Furthermore, when spatial factors were considered in Figure 11a,b,d, GDPP (IVA or PCO) imposed a positive externality on PM_2.5_; that is, the increase of GDPP (IVA or PCO) in one province may cause the increase of PM_2.5_ in the neighboring provinces. The reason is that the pollution particles, generated by the activities of residents, emissions from factories and private cars, may be passed from one province to the surrounding provinces through atmospheric movements such as wind speed, wind direction temperature. High temperature and wind speed can promote the convection of air. This can create better conditions for the dilution and dispersion of particulate matter. Notably, the Spearman’s rank correlation analysis and bivariate spatial correlation analysis gave a consistent conclusion for the downward trend on the UPD’s impact on PM_2.5_ concentration in Figure 6c and Figure 11c. It indicated that the impact of UPD on PM_2.5_ was getting smaller and smaller. This may be because of the population control policy. The population size of some provinces has been gradually controlled since the population control policy was implemented. The impact of UPD on PM_2.5_ may be closely related to population size [63].

Figure 12 showed the local bivariate cluster maps for PM_2.5_ and socioeconomic factors in 31 provinces of China, in 2016. For the provinces of the HH clusters, the development of the social economy in their adjacent provinces had positive radiation effect on these provinces. Their economic development in the local provinces have also brought about a number of pollution sources that have indirectly increased PM_2.5_. Some provinces with slow economic growth in Northwestern and Southwestern China had fewer pollution sources, which was easy to form LL clusters. Furthermore, the HL type provinces were mainly distributed in Xinjiang in Figure 10 and Figure 12. As a region of severe sandstorm and abundant coal resources, the exploitation and utilization of these coal resources have produced many pollutants in Xinjiang and destroyed the ecological balance of atmospheric environment. The complex topography of Xinjiang is also not conducive to the diffusion of atmospheric pollutants. In addition, Qinghai and Tibet with underdeveloped industry have less pollution resources and lower PM_2.5_ concentration. Rich vegetation in Sichuan and Yunnan can effectively reduce PM_2.5_ concentration. The combination of these factors formed the obvious HL outliers around Xinjiang.

### 4.3. The Spatial Spillover Effect of PM_2.5_

The spatial spillover effect means that the changes of PM_2.5_ concentration in one province can impact on PM_2.5_ concentration of other provinces. In this paper, spatial spillover effect of PM_2.5_ concentration in adjacent provinces can be reflected by the global Moran’s I. From Figure 9, there was a positive increasing trend of the global Moran’s I values of PM_2.5_ concentration during the study period. It indicated that the spatial correlation of PM_2.5_ gradually became stronger over time. The spatial autoregressive coefficient (W*PM25) were all significant (*p* < 0.01) in 1998–2016 (column 2 of Table 4). These meant that the spatial spillover effect is becoming more and more significant. From Figure 10 and Figure 12, the HH clusters of PM_2.5_ concentration (HH clusters of PM_2.5_ and socioeconomic factors) were mainly distributed in some provinces of economically developed area (i.e., North China, East China). At the same time, provinces of economically backward areas (i.e., Southwest China) appeared to have LL clusters. These indicated that the spatial spillover effect of North China, East China and Southwest China were higher than other regions. All the three regions have strong PM_2.5_ pollution homogeneity. In other words, there were spatial spillover effects in different provinces, but were particularly severe in North China, East China and Southwest China. However, we note that the spatial spillover effects on PM_2.5_ pollution for all regions are non-negligible. So local governments should consider the policies of adjacent provinces and coordination with adjacent provinces is indispensable.

### 4.4. Comparative Analysis of the Effects of GDPP, GDP per Area, IVA and IVA per Area on PM_2.5_

**Figure 13:**
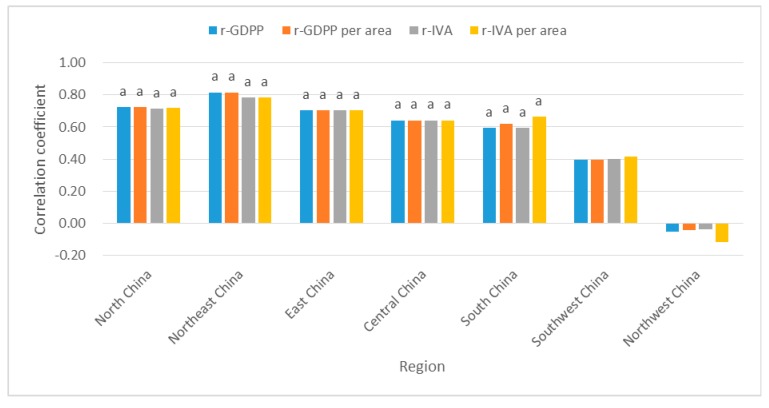
The correlation coefficient (r) values between PM_2.5_ and GDPP (IVA, GDPP per area or IVA per area) in the geographical subareas, 1998-2015. Notes: a, b represent coefficients significant at the 1%, 5% levels, respectively. No letters above the bar chart indicate insignificance.

To further analyze the correlation between PM_2.5_ and aerosol emission density, we used dataset of GDP per area and IVA per area to calculate the Spearman’s rank correlation coefficients. Due to the absence of data in 2016 and Tibet, the time sequence of the experiment was from 1998 to 2015, and Tibet was excluded. We have tested the correlations from a spatial perspective. The experiment was designed for the effects of GDPP, GDP per area, IVA and IVA per area on PM_2.5_ from two different scale, including regional scale and provincial scale. On regional scale, the correlation between PM_2.5_ and GDP per area (IVA per area, GDPP, or IVA) were significant in North China, Northeast China, East China, Central China and South China in Figure 13. On provincial scale, Table 5 showed that most provinces of the other five geographical regions except Southwest China and Northwest China presented a significant correlation between PM_2.5_ and GDP per area (IVA per area, GDPP, or IVA). Although there were some slight differences in the correlations values and p values under two different scales, the overall trend was consistent. These indicated that the increase of GDP and industry has a strong positive impact on PM_2.5_, especially in North China, Northeast China, East China, Central China and South China. However, the influence was not strong in Northwest China. The reason may be that PM_2.5_ concentration in Northwest China is more affected by sandstorms. These further validated the idea in this article. That is, human activities contribute to PM_2.5_ concentration, but are not the only factor.

## 5. Conclusions

This paper estimated spatial distribution, temporal variations and relationships of socioeconomic factors and PM_2.5_ in 31 provinces of China using a unary linear regression model, Spearman’s rank correlation analysis method, univariate spatial autocorrelation analysis method, bivariate spatial correlation analysis method and the spatial regression analysis during the period of 1998–2016. Results demonstrated that PM_2.5_ generally increased with the increase of socioeconomic factors from 1998 to 2016, but there were different temporal variations trend and relationships in different provinces and regions. Socioeconomic factors and PM_2.5_ concentration in most provinces in East China, South China, Central China, North China, and Northeast China had rapid growth trend, and socioeconomic factors were significantly correlated with PM_2.5_ concentration. Although the growth trend of socioeconomic factors in Southwest China and Northwest China were also fast, PM_2.5_ presented a slowly growth trend in Southwest China and a descending trend in Northwest China, and socioeconomic factors were weakly correlated with PM_2.5_ concentration. Urban population density was not an important influencing factor in affecting PM_2.5_ concentration. GDP per capita and industrial added values in the local and adjacent provinces were the key influencing factors for the increase of PM_2.5_ concentration. Private car ownership also contributed to PM_2.5_ concentration. PM_2.5_ in neighboring provinces were also an important factor to increase the local PM_2.5_ concentration. The results of the research can provide effective guidelines for urban sustainable development and further protect the environment of cities.

## Figures and Tables

**Table 1:** The values of GDP per capita (GDPP) (yuan/person), industrial added values (IVA) (billion yuan), urban population density (UPD) (person/per square kilometer), private car ownership (PCO) (10^4^ cars) and PM_2.5_ (μg/m^3^) in 1998 and 2016.

Region	1998					2016				
	PM_2.5_	GDPP	IVA	UPD	PCO	PM_2.5_	GDPP	IVA	UPD	PCO
East China	27.38	10,269.47	1269.72	1501.86	58.90	43.91	74,496.00	11,324.87	2756.00	5439.19
South China	16.32	7025.50	418.47	999.00	34.23	23.86	52,130.00	3995.00	2378.00	1943.85
Central China	30.62	4865.51	369.43	867.67	90.69	38.57	48,207.33	4091.64	3684.67	2056.22
Northwest China	35.26	4563.55	125.08	890.80	29.69	33.84	41,989.40	1398.92	2965.00	1090.65
Southwest China	16.19	3957.29	253.26	443.40	43.24	17.04	39,605.60	2493.59	2531.00	1904.52
North China	31.22	10,074.37	415.56	1338.40	125.75	45.28	76,781.80	3560.12	2634.60	2683.10
Northeast China	20.87	7591.06	350.02	722.00	41.11	32.38	48,363.67	1653.55	2986.67	1212.68
Mainland China	23.97	6860.00	3413.49	459.00	423.65	29.68	53,935.00	24,787.78	2408.00	16,330.22

**Table 2:** The variation trend of PM_2.5_, GDPP, IVA, UPD and PCO in the seven geographical subareas and Mainland China, 1998–2016.

Region	*z-slope*				
	PM_2.5_	GDPP	IVA	UPD	PCO
East China	0.129	0.174	0.175	0.162	0.165
South China	0.110	0.173	0.175	0.148	0.168
Central China	0.108	0.172	0.172	0.158	0.159
Northwest China	−0.015	0.172	0.171	0.136	0.160
Southwest China	0.066	0.170	0.170	0.156	0.163
North China	0.128	0.175	0.172	0.166	0.169
Northeast China	0.145	0.172	0.165	0.165	0.165
Mainland China	0.138	0.173	0.174	0.164	0.165

**Table 3:** Results of ordinary least squares regressions between PM_2.5_ and socioeconomic factors in Mainland China, 1998–2016.

Year	Variables									
	GDPP	IVA	UPD	PCO	R^2^	Log-L	SLM-LM	SLM-RLM	SEM-LM	SEM-RLM
1998	0.165	−0.0180	0.026	0.116	0.048	−42.717	5.571 **	1.583	4.826 **	0.838
1999	0.202	0.216	−0.012	−0.199	0.092	−41.981	2.778 *	0.115	2.667	0.005
2000	0.166	0.216	0.027	−0.076	0.085	−42.103	7.717 ***	0.587	7.222 ***	0.093
2001	0.361	0.168	0.068	−0.119	0.192	−40.179	7.661 ***	2.569	6.106 **	1.014
2002	0.438 **	0.422 *	−0.063	−0.292	0.302	−37.908	7.884 ***	0.850	7.036 ***	0.003
2003	0.449 **	0.460 *	0.195	−0.280	0.406	−35.417	8.874 ***	3.486 *	5.816 **	0.429
2004	0.466 **	0.470 *	0.144	−0.317	0.358	−36.611	6.037 **	2.729 *	3.933 **	0.625
2005	0.406 **	0.613 **	0.113	−0.414	0.363	−36.485	7.922 ***	1.938	6.067 **	0.082
2006	0.504 ***	0.384	0.265	−0.199	0.374	−36.216	13.218 ***	3.156 *	10.158 ***	0.096
2007	0.442 **	0.596 *	0.224	−0.354	0.390	−35.819	13.435 ***	3.741 *	9.842 ***	0.148
2008	0.546 ***	0.620 *	0.120	−0.429	0.429	−34.796	8.044 ***	2.218	5.844 **	0.018
2009	0.564 ***	0.494	0.139	−0.310	0.416	−35.141	7.249 ***	2.393	4.954 **	0.098
2010	0.439 **	0.515	0.515	−0.249	0.356	−36.665	6.853 ***	1.221	5.647 **	0.014
2011	0.501 ***	0.264	0.175	−0.012	0.379	−36.096	9.957 ***	2.826 *	7.183 ***	0.052
2012	0.424 **	0.236	0.144	0.082	0.336	−37.138	7.965 ***	3.252 *	5.070 **	0.356
2013	0.515 ***	0.013	0.201	0.229	0.371	−36.297	8.174 ***	4.133 **	4.726 **	0.685
2014	0.462 **	0.128	0.159	0.169	0.366	−36.422	6.566 **	4.074 **	3.335 *	0.842
2015	0.575 ***	−0.019	0.226	0.221	0.419	−35.051	6.294 **	3.891 **	3.196 *	0.793
2016	0.578 ***	−0.286	0.122	0.494	0.385	−35.939	5.762 **	5.050 **	2.465	1.752

Notes: Log-L denotes Log likelihood; *, **, ***represent coefficients are significant at the 10%, 5%, 1% levels, respectively.

**Table 4:** The results of spatial lag model regression in 1998–2016.

Year	Variables								
	W*PM25	GDPP	IVA	UPD	PCO	R^2^	Log-L	AIC	SC
1998	0.646 ***	0.151	0.005	−0.114	0.064	0.328	−39.165	90.330	98.934
1999	0.488 ***	0.196	0.149	−0.110	−0.156	0.237	−40.237	92.474	101.078
2000	0.677 ***	0.087	0.170	−0.121	−0.057	0.401	−37.598	87.197	95.800
2001	0.694 ***	0.245	0.095	−0.128	−0.098	0.483	−35.457	82.914	91.518
2002	0.674 ***	0.279 **	0.258	−0.191	−0.233	0.549	−33.196	78.391	86.995
2003	0.647 ***	0.225 *	0.345 *	0.082	−0.262	0.618	−30.406	72.812	81.416
2004	0.595 ***	0.274 *	0.345 *	0.046	−0.315	0.536	−33.075	78.150	86.754
2005	0.623 ***	0.206	0.476 **	0.028	−0.422 *	0.570	−32.075	76.151	84.755
2006	0.766 ***	0.227 **	0.378 *	0.274 ***	−0.311	0.690	−28.214	68.428	77.032
2007	0.754 ***	0.207 *	0.471 **	0.217 **	−0.407 *	0.695	−27.832	67.664	76.268
2008	0.646 ***	0.311 **	0.535 **	0.121	−0.483 **	0.630	−29.898	71.796	80.400
2009	0.645 ***	0.321 **	0.502 **	0.144	−0.447 *	0.614	−30.556	73.112	81.716
2010	0.626 ***	0.242 *	0.515 *	0.153	0.383	0.561	−32.421	76.842	85.446
2011	0.729 ***	0.238 **	0.369	0.186 *	−0.268	0.653	−29.580	71.160	79.764
2012	0.674 ***	0.206	0.290	0.176	−0.128	0.580	−32.073	76.146	84.750
2013	0.679 ***	0.244 *	0.223	0.204 *	−0.098	0.607	−31.099	74.198	82.802
2014	0.637 ***	0.203	0.302	0.166	−0.137	0.571	−32.146	76.292	84.896
2015	0.639 ***	0.266 **	0.249	0.175	−0.148	0.606	−30.845	73.689	82.293
2016	0.628 ***	0.247 *	0.081	0.107	0.06	0.570	−32.100	76.201	84.805

Notes: Log-L denotes log likelihood; *, **, ***represent coefficients significant at the 10%, 5%, 1% levels, respectively.

**Table 5:** The correlation coefficients of provinces in seven geographical subareas, 1998–2015.

Region	Province	Correlation Coefficient (r)			
		r-GDPP	r-GDPP per Area	r-IVA	r-IVA per Area
**North China**	Beijing	0.785 **	0.777 **	0.779 **	0.779 **
	Tianjin	0.701 **	0.701 **	0.695 **	0.695 **
	Hebei	0.756 **	0.756 **	0.759 **	0.759 **
	Shanxi	0.525 *	0.525 *	0.548 *	0.453
	Inner Mongolia	0.323	0.342	0.311	0.443
**Northeast China**	Liaoning	0.822 **	0.812 **	0.783 **	0.783 **
	Jilin	0.829 **	0.829 **	0.822 **	0.822 **
	Heilongjiang	0.742 **	0.740 **	0.600 **	0.600 **
**East China**	Shanghai	0.604 **	0.604 **	0.556 *	0.556 *
	Jiangsu	0.754 **	0.754 **	0.754 **	0.754 **
	Zhejiang	0.560 *	0.558 *	0.560 *	0.548 *
	Anhui	0.798 **	0.804 **	0.798 **	0.798 **
	Fujian	0.474 *	0.474 *	0.474 *	0.474 *
	Jiangxi	0.552 *	0.548 *	0.554 *	0.439
	Shandong	0.752 **	0.752 **	0.752 **	0.750 **
**Central China**	Henan	0.756 **	0.756 **	0.773 **	0.763 **
	Hubei	0.641 **	0.641 **	0.628 **	0.628 **
	Hunan	0.585 *	0.585 *	0.585 *	0.585 *
**South China**	Guangdong	0.552 *	0.552 *	0.552 *	0.552 *
	Guangxi	0.649 **	0.626 **	0.649 **	0.484 *
	Hainan	0.498 *	0.643 **	0.513 *	0.628 **
**Southwest China**	Chongqing	0.331	0.331	0.340	0.340
	Sichuan	0.418	0.418	0.449	0.480 *
	Guizhou	0.467	0.467	0.467	0.488 *
	Yunnan	0.457	0.515 *	0.449	0.368
**Northwest China**	Shaanxi	−0.030	−0.028	−0.003	−0.096
	Gansu	–0.152	0.038	−0.160	0.189
	Qinghai	0.567 *	0.451	0.579 *	0.480 *
	Ningxia	−0.562 *	−0.562 *	−0.562 *	−0.470 *
	Xinjiang	0.240	0.240	0.230	0.232

Notes: *, ** represent coefficients are significant at the 5%, 1% levels, respectively.
